# Copland on Fever

**Published:** 1848

**Authors:** James Copland


### ARTICLE IX.

*On Fever.* By James Copland M.D. F.R.S., &c., &c.

[A very interesting question arises in the study of fever, as to the manner in which the blood and the various organs become affected. Is the morbific influence conveyed to the secreting and other organs by means of the blood or of the organic nervous system, or by mere continuity and contiguity of surface and structure? And is the blood itself affected by the direct contact of a poison, or by nervous influence, or by failure of excreting processes? Dr. Copland shall answer. He observes,]

It would be interesting to trace the manner in which the different systems of the frame become affected during the progress of various fevers. There is a certain class, for instance, that has been called periodic fevers; others, typhoid fevers; and others pestilential or malignant fevers. Fevers have been variously classed; indeed they admit of a variety of classifications. With respect to periodic fevers, or those that arise from malaria, it may be remarked, that the morbid influence producing them seems to implicate especially the organic nervous system. There are various reasons for believing that this system is primarily affected, and that the fluids and abdominal viscera become more or less disordered secondarily. These reasons appear to be, first, the long period of incubation in many of the periodic fevers. Intermittent fever may not appear until many weeks after the individual has been exposed to malaria, and then the paroxysms occur at intervals. We know that morbid conditions affecting nervous parts, usually assume a periodic character. If it were the blood which is affected in these cases, we might infer that that state of the blood which existed during the paroxysm would most likely continue without intermission until removed: and that, instead of an interval, when comparitively little disorder is felt, the disease would be continued; for we observe that, in proportion as the blood becomes affected in fever, so does the disease assume a more and more continued type. There are various lesions prominently affecting particular organs, and which give fevers variety of character: thus we have gastric fever, billious fever, and so on.

If we proceed to the consideration of the worst forms of fever, for instance, typhus, or pestilential fever, not only is the nervous system affected---the organic nervous system being probably the first to be impressed with the cause or the disease---but also the blood itself soon becomes more or less disordered---becomes physically changed.

Now the question is, whether this change produced so early in the blood in typhoid and pestilential fevers, arises primarily from the absorption of the cause into the blood, or whether it arises from the morbid impression made on the organic nervous system, owing to which impression the excreting or depurating functions, which are under the influence of this system, are impaired or arrested, and the circulating vessels become affected, and ultimately changed? I may point out to you how copiously the vascular system is supplied by the organic or ganglial system; and hence we may expect, *a priori*, that causes affecting this system will, to a co-ordinate extent, affect the vascular system and the fluids in the vessels. We find that, in the progress of fevers, the blood becomes changed; and the change may arise partly from the impression made by the emanations causing the fever, upon the organic nervous system, and partly from the absorption of the emanation---of the morbid poison---into the circulating mass. I believe that the morbid effluvium, being received into the lungs with the air, injuriously affects the organic nervous system supplying these organs: hence the blood in the lungs is not sufficiently changed. Possibly partial absorption of this effluvium may take place into the blood itself; but if it does not, there is a still stronger reason to infer that the morbid impression extends throughout the organic nervous system, impairing the influence of it upon the vascular system, and in the secreting and excreting viscera. The organic nervous power being depressed, it naturally follows that the organs supplied by this system will be impaired in function; and hence we find that, in the first days, or within the first twenty-four hours of fever, the functions of the excreting organs are very remarkably diminished, or even suppressed; and the consequence is, that the blood, which may have been hitherto unaffected, becomes changed; or, if it have been already affected by the cause of the disease, or by the impression of the ganglial system, becomes still further changed. Thus the one change reacts on the other, and promotes it, until at last the changes in the organic or vital nervous influence, and in the vascular system and circulating fluids, become so great, that the blood is not only altered sensibly as it respects both its physical characters and its chemical qualities, but the tissues and organs themselves become more and more disorganized, or evince a remarkable loss of their natural vital cohesion, especially in distempers of a malignant character.—*Med. Gazette* in Braith's Ret.

